# Protective Effect of *Costus afer* Aqueous Leaf Extract (CALE) on Low-Dose Heavy Metal Mixture-Induced Alterations in Serum Lipid Profile and Hematological Parameters of Male Wistar Albino Rats

**DOI:** 10.1155/2020/8850264

**Published:** 2020-09-29

**Authors:** Brilliance O. Anyanwu, Chinna N. Orish, Anthonet N. Ezejiofor, Ify L. Nwaogazie, Orish E. Orisakwe

**Affiliations:** ^1^African Centre of Excellence for Oilfield Chemicals Research (ACE-CEFOR), University of Port Harcourt, PMB 5323, Port Harcourt, Choba, Nigeria; ^2^Department of Anatomy, Faculty of Basic Medical Sciences, College of Health Sciences, University of Port Harcourt, PMB 5323, Port Harcourt, Choba, Nigeria; ^3^African Centre of Excellence for Public Health and Toxicological Research (ACE-PUTOR), University of Port Harcourt, PMB 5323, Port Harcourt, Choba, Nigeria

## Abstract

The present work investigated the protective effects of *Costus afer* Ker Gawl. aqueous leaf extract (CALE) on lipid profile and hematological changes induced by exposure to low-dose heavy metal mixture in male albino rats. The experimental animals were divided into six weight matched groups. The normal (group 1) and toxic (group 2) controls received deionized water and metal mixture (20 mg/kg PbCl_2_, 1.61 mg/kg CdCl_2_, and 0.40 mg/kg HgCl_2_), respectively. Test rats in groups 3, 4, and 5 were treated with metal mixture and CALE (750, 1500, and 2250 mg/kg, respectively), and group 6 received metal mixture and ZnCl_2_. All treatments were administered through oral gavage for 12 weeks. LDHMM caused a marked increase (*p* < 0.05) in cholesterol, triglyceride, low-density lipoprotein (LDL), and very low-density lipoprotein (VLDL) levels and a decrease in high-density lipoprotein (HDL), percentage body weight gain, and feed and fluid intake. Also, a significant decrease in RBC, Hb, and PCV, a significant increase in WBC, and no significant increase in platelet PLT were observed in the metal mixture-treated group. But in CALE treated groups, their levels were found to attain almost normal values as found in normal control which is also similar to the zinc-treated group. *Costus afer* may hold a promise in improving lipid profile and hemodynamic picture in cardiovascular diseases.

## 1. Introduction

The world's principal causes of mortality have been associated with cardiovascular-related diseases [1], and in effect, a regular test on the serum lipid profile is vital to calculate the risk connected with the cardiovascular system [2]. Cardiovascular risk problems could result from heavy metal exposure in our surroundings or workplace [3]. The liver, kidney, lung, heart, testis, and blood system have been listed among the parts of the body that are affected by Pb, Cd, and Hg toxicity [2, 4]. A recent report by Salazar-Flores et al. [5] revealed that heavy metals lead to oxidative stress which causes tissues and membranes degeneration in a living organism, and because of this property (its capacity to scavenge ROS), antioxidants are needed to protect against its toxicity.

Human and animal health care treatment with ethnomedicine is no longer seen as a myth or unholy practice [6, 7] and this has gained popularity in different parts of the world. It is recently believed that “natural is better” and approximately 80% of world's population depends solely on herbal medicines for the treatment of diseases [6, 7]. Owing to the fact that these herbal medicines are constantly available, simply affordable, and already an essential part of the people's way of life, the World Health Organization (WHO) encourages developing countries to make use of herbal medicine in treating health problems affecting them locally [6].

The use of traditional medicine has been essential to the health of millions of people resident in rural and semiurban regions of African countries, where these herbal remedies are mostly available and simply affordable [8, 9]. *Costus afer* (Ker Gawl.) is a herbaceous plant that belongs to the family Zingiberaceae and has been evidenced to have many therapeutic effects in humans and animals. Reports on the phytochemical analysis of *Costus afer* revealed that the plant is rich in steroidal saponins, flavonoids, alkaloids, tannins, terpenoids, sapogenins, oxalates, furans, furan derivatives, and starches [10].These phytochemicals are rich in antioxidants. The pharmacological activities associated with *Costus afer* include antioxidant property, hepatoprotective, nephroprotective, antidiabetic role [11, 12], and antinociceptive role [13].

There is little knowledge on *Costus afer* effects on low-dose heavy metal mixture (Pb, Cd, and Hg) induced serum lipid profile alterations and hematological changes despite its known pharmacological benefits. Over ten years ago, traditional medicines have become a topic of global discussion and many developing nations depend on this type of medication to solve their health care problems [6]. *Costus afer* is commonly found in shady or moist forests or riverbanks of tropical West Africa which includes Cameroun, Ghana, and Nigeria and has been beneficial to many cultures in treating diseases. This research evaluated the effect of *Costus afer* aqueous leaves extract (CALE) on low-dose heavy metal mixture- (LDHMM-) mediated serum lipid profile and hematological alterations in male albino rats.

## 2. Materials and Methods

### 2.1. Collection and Identification of *Costus afer*

*Costus afer* was harvested from the interior part of a farm land (away from vehicular traffic) behind the World Bank African Centre of Excellence for Oilfield Chemicals Research, University of Port Harcourt in Ikwerre Local Government Area of Rivers State, Nigeria. Identification and authentication of the plant material were done by Mr. A.O. Ozioko and given the voucher number (INTERCEDDO/033).

### 2.2. Preparation of the Plant Extract

Fresh leaves of *Costus afer* were collected and washed clean to eliminate any form of contamination. The leaves were pulverized and properly stored. Then, 250 g of the pulverized leaves was weighed and macerated in 500 ml deionized water in a stoppered container and allowed to stand for 24 hours with constant agitations at intervals following the previous works of Ezejiofor and Orisakwe [12]. After vigorous shaking of the mixture, the pulverized leaves were pressed and the extract was separated. The filtrate was then stored in a refrigerator at 4°C. The extract was redundant after the fourth day of treatment and fresh preparation was made. This process was continuous over the 90 days of treatment.

### 2.3. Animal Care Handling

Young male Wistar rats, approximately 8 weeks old and weighing 100–200 g, bought from the animal house of the Department of Experimental Pharmacology and Toxicology, University of Port Harcourt, Choba, Rives State, Nigeria, were used for the study. The test animals were kept for fourteen days to adapt in polypropylene cages under standard conditions [12]. The protocol for the experiment was approved by the University of Port Harcourt Research Ethics Committee and the reference number UPH/CEREMAD/REC/04 was assigned. The animals were given standard feed and deionized water *ad libitum*.

### 2.4. Experimental Design

Weight matched rats were divided into six groups of seven rats each. Group 1 or the control received only the deionized water, while group 2 received heavy metal mixture only (PbCl_2_, 20 mg/kg; CdCl_2_, 1.61 mg/kg; HgCl_2_, and 0.40 mg/kg) (Sigma Aldrich WGK, Germany) according to the study by Institóris et al. [14]. Rats in groups 3, 4, and 5 received the heavy metal mixture and *Costus afer* extract at 750 mg/kg, 1500 mg/kg, and 2250 mg/kg, respectively, according to Ezejiofor and Orisakwe [12].

### 2.5. Sample Collection

After the 12 weeks of treatment, rats were sacrificed under ether anesthesia. Approximately, 4 ml of blood was taken from each rat by cardiac puncture, divided into two parts, and stored in EDTA and plain vial for hematological and biochemical analysis, respectively. The serum was collected in fresh clean tubes after centrifugation (3000 rpm at 2°C for 15 minutes) and stored at −20°C for lipid profile assay following a study by Sharma and Kumari [2].

### 2.6. Heavy Metal Analysis on Blood Samples

The frozen blood samples of both the control and experimental animals were retrieved and allowed to thaw. An aliquot of 1 ml blood was drawn using a micropipette into clean test tube. Two millilitres (2 ml) of concentrated nitric acid (HNO_3_) was added and left over to digest the blood samples. The solution was later made up to 25 ml with deionized water. Solar thermoelemental flame atomic absorption spectrometer (Model SG 71906) was used to determine the blood levels of lead, cadmium, and mercury.

### 2.7. Estimation of Lipid Profile

Commercial kits (Randox Laboratories Ltd., UK) were used to assay the concentrations of cholesterol, high-density lipoprotein (HDL), and triglycerides in serum assay. The concentration was calculated as

(1) $$\begin{array}{c} \text{Conc}.\text{ sample }=\frac{\Delta A\text{ sample }\times \text{conc}.\text{ standard}}{\Delta A\text{ standard}}, \end{array}$$

where Δ*A* represents change in absorbance and conc. represents concentration.

Friedewald's equation [15] was used to estimate the levels of LDL and VLDL:

(2) $$\begin{array}{c} \text{LDL}=\left[\text{TC}\right]-\left[\text{HDL}\right]-\frac{\left[\text{TG}\right]}{2.2},\\ \text{VLDL}=\frac{\text{triglyceride}}{2.2}. \end{array}$$

### 2.8. Hematological Parameters

The volume of blood cells present in the whole blood sample was analyzed by using an automated haematology analyzer (MY-B003, China). The result showed the concentrations of red blood cells, white blood cells, platelets, haemoglobin, and packed cell volume, among others, in the whole blood sample.

### 2.9. Statistical Analysis

In this study, statistical data analysis attempted to capture three major areas of emphasis. The first is the descriptive statistics for which the mean and standard deviation of all the parameters were computed. The second is the analysis of variance (ANOVA) applied to the sequence of observations for the purpose of comparative analysis at 5% significance. This approach is known as inferential statistics wherein a null hypothesis can be accepted or rejected given the outcome of ANOVA test, significant or not. For this study, the multiple comparisons were carried out with Duncan's multiple comparison method at 5% significant level. The third is establishing a relationship between “cause and effect,” that is, independent variable(s) as “cause” and dependent variable as “effect.” In reality, the number of independent variables may be large for a multiple regression. Thus, the principal component analysis (PCA) was adopted to help select the principal factors (or independent variables) for developing the multiple regression equations. In this study, the use of PCA was accomplished using XLSTAT 2016 [16].

## 3. Results

### 3.1. Evaluation of Body Weight Difference

**Figure 1:**
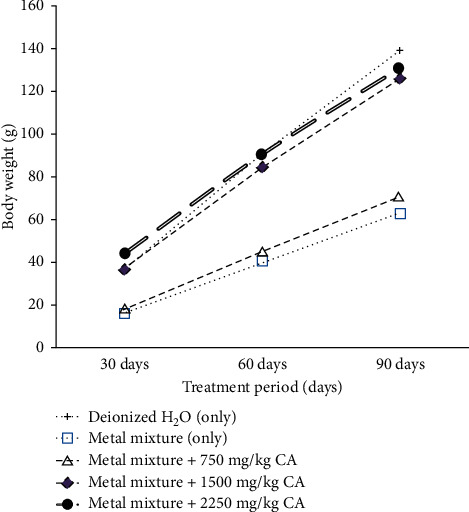
Effect of *Costus afer* on body weight of male albino rats exposed to heavy metal mixture.

The effect of *Costus afer* on the body weight of rats exposed to heavy metal mixture is shown in Figure 1. The final body weight of the heavy metal mixture only treated group was significantly lower than that of the control group. The final body weights of the metal mixture + 750 mg/kg *Costus afer*, metal mixture + 1500 mg/kg *Costus afer*, and metal mixture + 2250 mg/kg *Costus afer* groups showed an increase in body weight gain.

### 3.2. Evaluation of the Feed Efficiency and Feed and Fluid Intakes

Table 1 shows the effects of *Costus afer* on the feed efficiency and feed and fluid intakes of rats exposed to heavy metal mixture. After 12 weeks of treatment, there was a significant decrease in the feed and fluid intake of rats that received the heavy metal mixture only compared to the rats treated with only deionized water in the control group. Cotreated rats with *Costus afer*, however, showed a significant increase in their feed and fluid intake. The feed and fluid intakes of rats treated with heavy metal mixture (98.23 ± 11.23, 306.46 ± 20.8, *p* < 0.05), respectively—at the end of 12 weeks period—were statistically different from those cotreated with *Costus afer* at varying doses. This effect shown by rats in the heavy metal treated group was also significantly different from the effects shown by rats treated with deionized water only (226.36 ± 5.43, 439.45 ± 8.50, *p* < 0.05), respectively. The effect shown in rats cotreated with *Costus afer* showed a dose-dependent effect.

### 3.3. Hematological Assay

The hematological effect of *Costus afer* on the rats exposed to heavy metal mixture is shown in Table 2. A significant decrease (*p* < 0.05) in RBC, Hb, and PCV, a significant increase in WBC, and no significant increase in PLT were observed in the metal mixture treated animals compared to the control rats. The RBC, Hb, PCV, WBC, and PLT of rats treated with the heavy metal mixture (3.1 ± 0.15, 8.6 ± 1.07, 25.2 ± 3.14, 15.0 ± 2.02, and 219.0 ± 43.70, *p* < 0.05), respectively, were significantly different from rats cotreated with *Costus afer* at varying doses. The effect shown in rats cotreated with *Costus afer* showed a dose-dependent effect.

### 3.4. Lipid Profile Assay

**Figure 2:**
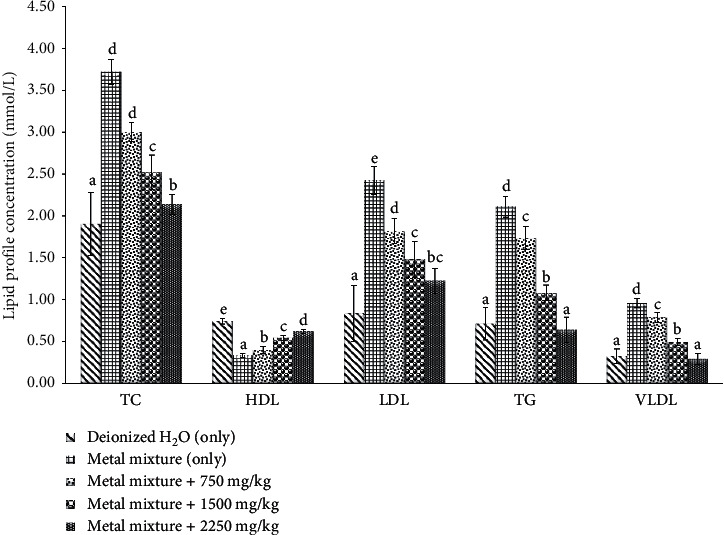
Effect of *Costus afer* on lipid profile parameters of male albino rats treated with metal mixture.

The cholesterol, HDL, triglycerides (TG), LDL, and VLDL levels in heavy metal mixture exposed rats with or without *Costus afer* treatment aare shown in Figure 2. The cholesterol, HDL, TG, LDL, and VLDL levels in rats treated with heavy metal mixture (3.72, 0.336, 2.11, 2.42, and 0.96, *p* < 0.05), respectively, were significantly different from rats cotreated with *Costus afer*. The effect shown in rats cotreated with *Costus afer* showed a dose-dependent effect.

**Figure 3:**
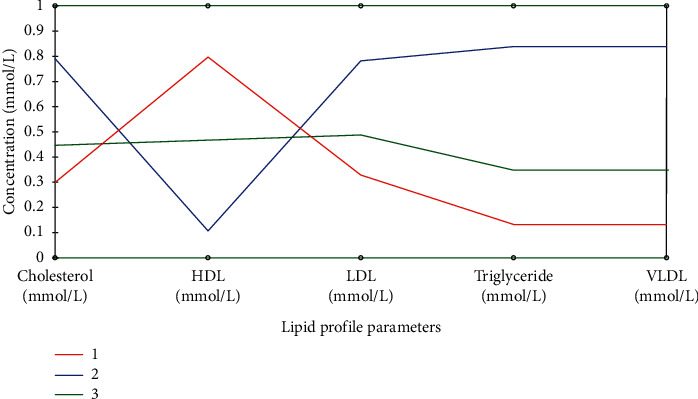
A parallel coordinates plot showing clustering of lipid profile interaction in different groups.

**Figure 4:**
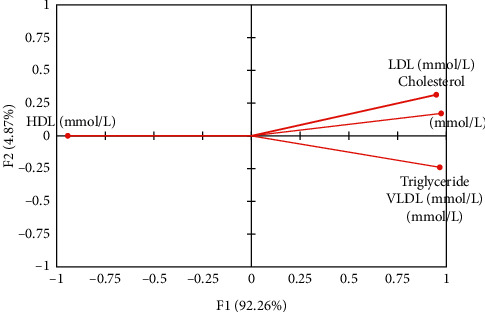
Correlation plot of lipid profile parameters on male albino rats against the generated factors (F1 and F2). Variables (axes F1 and F2: 97.13%).

**Figure 5:**
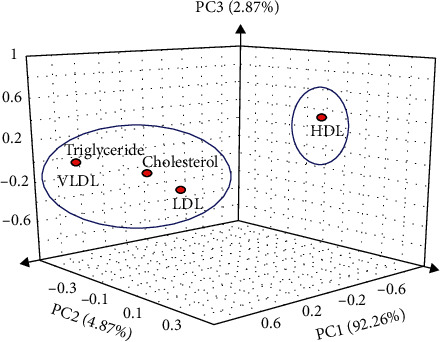
Score plot illustrating the differentiation of parameters associated with interactions among lipid profile parameters. A three-component system explaining 100% of total variance was observed after statistical PC analysis.

A parallel coordinates plot of lipid profile parameters showing the relationship between the variables is presented in Figure 3. The plot comprises three classes; class 1 which include rats in groups 1 and 5, class 2 comprising rats in groups 2 and 3, and class 3 which include rats in group 4.  Table 3 shows the factor loading of lipid profile variables on significant principal components after Varimax rotation, while Figure 4 presents the correlation plot. Score plot illustrating the differentiation of parameters associated with interactions among lipid profile parameters in different groups is depicted in Figure 5. A three-component system explaining 100% of total variance was observed after statistical principal component analysis.

### 3.5. Evaluation of the Levels of Lead (Pb), Cadmium (Cd), and Mercury (Hg) in Blood

**Figure 6:**
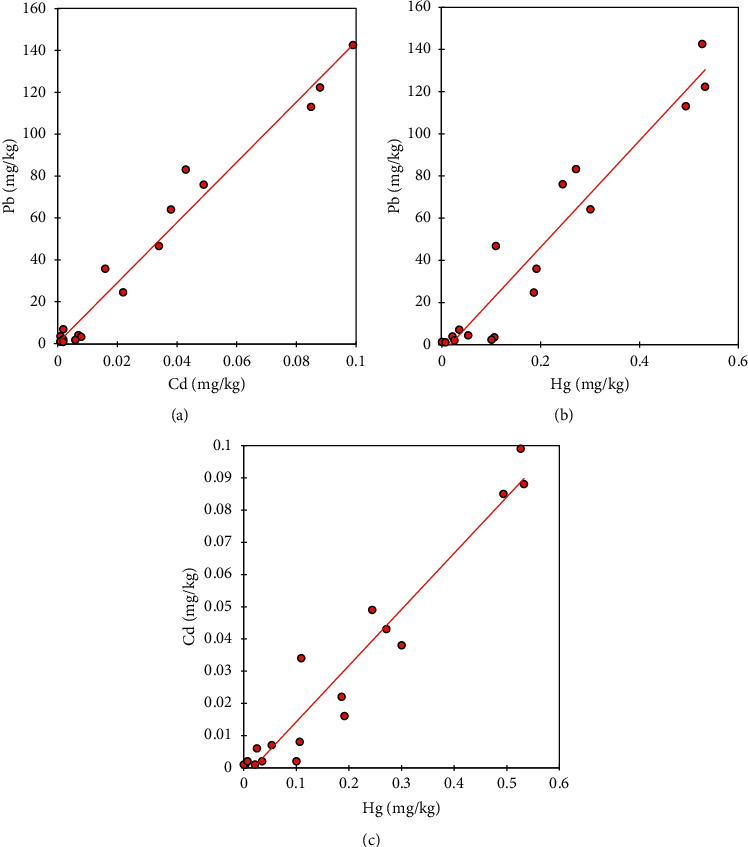
Interelemental correlation among toxic metals within blood of rats showed strong positive correlation (*r* > 0.90) between metals such as (a) Cd and Pb, (b) Hg and Pb, and (c) Hg and Cd during the study. All correlations were significant at *p* < 0.01.

The concentrations of Pb, Cd, and Hg in blood tissue were markedly increased (*p* < 0.05) in heavy metal mixture-treated rats compared to those in control rats (Table 4). Coadministration with *Costus afer*, however, significantly decreased accumulation of the heavy metals in the blood. The metal mixture intoxicated rats were the most concentrated (Pb = 125.901 ± 15.069, Cd = 0.091 ± 0.007, and Hg = 0.518 ± 0.021) compared to the control group (treated with deionized water only). Pearson's rank correlation showed the interelemental relationship between toxic metals with positively strong correlation (*r* > 0.90) between metals (a) Cd and Pb, (b) Hg and Pb, and (c) Hg and Cd during the study (Figure 6).

### 3.6. Model Development

**Figure 7:**
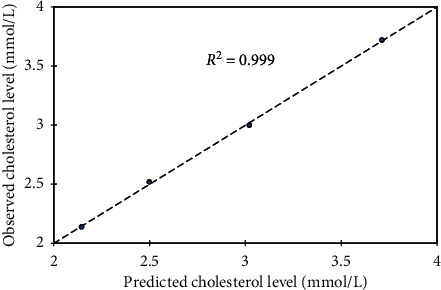
Predicted total cholesterol level against observed one as a lipid profile parameter, where *y* = total cholesterol levels and *x* = *Costus afer* concentrations served as input values.

**Figure 8:**
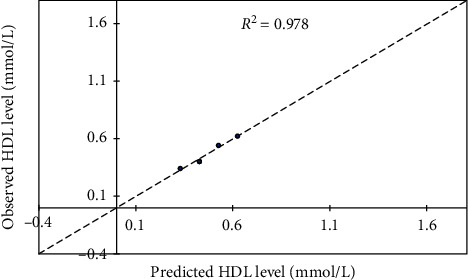
Predicted HDL level against observed one as a lipid profile parameter, where *y* = HDL levels and *x* = *Costus afer* concentrations served as input values.

**Figure 9:**
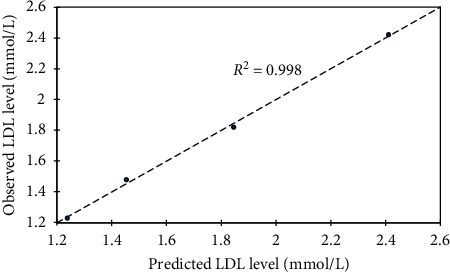
Predicted LDL level against observed one as a lipid profile parameter, where *y* = LDL levels and *x* = *Costus afer* concentrations served as input values.

**Figure 10:**
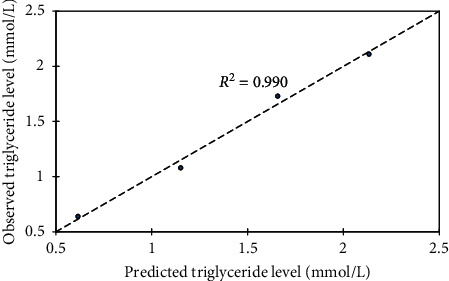
Predicted triglyceride level against observed as a lipid profile parameter, where *y* = triglyceride levels and *x* = *Costus afer* concentrations served as input values.

A statistical tool, XLSTAT 2016 (version 6 statistical package), was used to develop the models. Firstly, four independent parameters (Pb, Cd, Hg, and *Costus afer*) formed the input data for the multiple linear regressions in order to calibrate the model. The output indicates zero coefficients for *x*_2_ and *x*_3_. Thus, subsequent model calibration excluded the three constant parameters which formed the input data. In effect, *y* becomes a function of *Costus afer* variable for a given set of Pb, Cd, and Hg variables. Subsequently, concentration of total cholesterol (*y*) against *Costus afer* (*x*) was subjected into trial models of linear, quadratic, and exponential options (Table 5). A repetition was done for other parameters and the best with respect to goodness of fit (*R*^2^), mean square error (MSE), and root mean square error (RMSE) values were selected and summarized as shown in Table 6 for all lipid profile experiments. For model verification, it was imperative to compare the observed against predicted total cholesterol level (Figure 7) with corresponding goodness of fit, *R*^2^ = 0.999. Similarly, the verification was done for other lipid profile parameters (Figures 8–10). Hence, each model can be useful in forecasting the applicable dependent variable for a given independent variable (*Costus afer*) at constant Pb, Cd, and Hg concentrations as used in this current research.

A repetition of regression models was carried out on other parameters and the best models with respect to *R*^2^, MSE, and RMSE values were selected and summarized as shown in Table 6.

## 4. Discussion

The final body weight of rats treated with heavy metal mixture was significantly lower than those treated with only the deionized water. In contrast, the final body weight of rats cotreated with *Costus afer* at 750 mg/kg, 1500 mg/kg, and 2250 mg/kg was statistically higher (*p* < 0.05) than the heavy metal mixture treated rats. According to Lu et al. [17], a direct evidence of toxic injury in laboratory models is frequently signified by a decrease in body weight. A decrease or an inhibition in body weight gain may be seen in an organism after exposure to heavy metals [12]. The decrease in body weight gain may be caused by failure of appetite for food and drink which is evidenced by the result of the feed and fluid intake. However, supplementation with *Costus afer* resulted in an increase in body weight gain which is similar to the normal rats. Therefore, *Costus afer* may inhibit the weight loss caused by low dose heavy metal mixture exposure. A reduction in feed and fluid intake was obvious in animals treated only with the toxicants compared to the rats given deionized water. This observation is in line with the study of Nakade et al. [18]. Obviously from the result, a decrease in appetite for water and food may be a result of heavy metal poisoning. This also can be related to the difference in body weight shown by rats treated with only the metal mixture. Cotreated rats with *Costus afer*, however, showed a rise in their feed and fluid intake.

There was a considerable reduction (*p* < 0.05) in RBC, Hb, and PCV, a marked increase in WBC, and nonsignificant increase in PLT noticed in the metal mixture treated rats compared to the deionized water treated rats. The changes serve as the earliest indicators of toxic effects [19]. Similar findings have also been detailed by Ezejiofor and Orisakwe [12]. According to Kenston et al. [20], these results could cause anemia, coagulation disorders, and other hemorrhagic conflicts. Contradictory to the observation from heavy metal mixture group, the groups cotreated with *Costus afer* exhibited their protective effect by influencing the changes resulting from heavy metal mixture exposure and this implies that *Costus afer* has the potential to mitigate anemia and other blood related diseases comparable to an essential trace element like zinc.

Investigating the lipid profile is essential to avoid problems related to cardiovascular system of the body. The heavy metal mixture disturbed the serum lipid content (cholesterol, HDL, triglycerides, LDL, and VLDL). This is obvious by an increase in total cholesterol, triglycerides, LDL, and VLDL concentrations with a decrease in HDL. This finding is similar to the previous work of Kaur and Sharma [21]. In rats cotreated with *Costus afer*, the triglyceride was decreased as high density lipoprotein (HDL) increased. *Costus afer* reduced triglyceride which may interpret that they increase lipase activity which hydrolyzes lipids. The changes recorded in animals treated with only the toxicants are indicators of hyperlipidemia and diabetes mellitus [22, 23], which is susceptible for coronary heart disease. The rise in triglyceride content could be the result of hypoactivity of lipoprotein lipase in blood vessels which may explain impaired triglycerides metabolism and higher triglyceride levels [2]. The relationship between low HDL level and high triglyceride has been reported by Tersawa et al. [24] and Sharma and Kumari [2] leading to high probability of atherosclerosis. The concentrations of LDL and VLDL were considerably high in metal mixture treated group in comparison to other groups. These results are akin to the reports of Larregle et al. [25]. Their increase could possibly be due to reduced efflux of circulatory VLDL cholesterol particles as a result of decreased lipoprotein lipase (LPL) activity, leading to decreased LDL catabolism and increased LDL levels [26]. The changes in lipid profile contents recorded in rats exposed to only the metal mixture were protected by cotreatment with the plant extract. This proves the protective efficacy of *Costus afer* against heavy metal mixture (Pb, Cd, and Hg) induced damage to serum lipid profile. This finding proposes that the *Costus afer* may be useful in keeping at woof the probability of cardiovascular diseases.

A parallel coordinates plot of lipid profile parameters showing the relationship between the variables comprises three classes: class 1 which includes rats in groups 1 and 5, class 2 comprising rats in groups 2 and 3, and class 3 which include rats in group 4. This shows that class 1 rats have low TC, TG, LDL, and VLDL levels with increased HDL, while class 2 rats have high TC, TG, LDL, and VLDL levels with reduced HDL. Class 3 rats fit in to the intermediate class implying that they have in-between values for all parameters.

Three principal factors (F1–F3) were extracted explaining 100% cumulative variations in the lipid profile parameters. Factor 1 (F1) had eigenvalue > unity explaining 92.26% of total variation, while factors 2 and 3 (F2 and F3) had eigenvalues < unity explaining 7.74% of the total variation. Varimax rotation was executed on F1 and F2 to improve the PCA result. Correlated coefficients are printed in bold prints and are markedly significant for the rotated components. Variables clustered together around the axes indicate component factor values with the highest correlation coefficient (such as triglyceride and VLDL). Variables that form an acute angle have very strong positive correlation between them (such as LDL and TC), while variables at right angle have weak or no correlation between them. Also, any two variables that are opposite themselves have inverse relationship between them (such as HDL and TC, TG, LDL, and VLDL). This signifies that a reduction in HDL results in an increase in TC, TG, LDL, and VLDL and vice versa. Given that all the variables had high loadings on PC1 after Varimax rotation, the principal components were extracted and using a 3D graphing software, the correlated variables were plotted.

The considerable reduction in the blood levels of lead (Pb), cadmium (Cd), and mercury (Hg) following the administration of *Costus afer* is noteworthy. Although the exact mechanism is unclear but chelation of these metals by an active ingredient of this natural antidote is obvious. Recent developments in oxidative stress and understanding of its management require a dual function of the antioxidants, namely, metal chelation and free radical scavenging [27, 28]. The above proven antioxidant mechanism of *Costus afer* suggests the presence of metal ion chelating activity of an antioxidant moiety which prevents oxyradical generation and consequent oxidative damage [29]. Further studies will be needed to understand the exact mechanism of action and the levels of these metals in urine and fecal matter to ascertain their routes of elimination.

There were very strong significant correlations (*r* > 0.90) between Cd–Pb (*r* = 0.986, *p* < 0.05, *n* = 18), Hg–Pb (*r* = 0.963, *p* < 0.05, *n* = 18), and Hg–Cd (*r* = 0.967, *p* < 0.05, *n* = 18) in blood. The strong correlations seen among these toxic metals indicate a close physiological relationship [30]. The strongest correlation was observed in the association between blood Cd and blood Pb. This finding could be as a result of similar oxidative states of these toxic metals, which make them exhibit analogous chemical properties.

The linear and exponential models for total cholesterol had *R*^2^, MSE, and RMSE of 0.978, 0.015, and 0.122 and 0.995, 0.003, and 0.055, respectively. The 2^nd^-order polynomial model was the best model with respect to goodness of fit; *R*^2^, mean square error (MSE), and root mean square error (RMSE) values (i.e., 0.999, 0.001, and 0.031). A repetition of the regression models was done for other lipid profile parameters (low-density lipoprotein (LDL), triglycerides (TG), and high density lipoprotein (HDL)) and based on *R*^2^, MSE, and RMSE, the best models were selected. The *R*^2^, MSE, and RMSE of linear, exponential, and polynomial models for HDL were 0.978, 0.001, and 0.023; 0.979, 0.001, and 0.024; and 0.980, 0.001, and 0.031, respectively, and the linear model was selected with respect to its *R*^2^, MSE, and RMSE values. The *R*^2^, MSE, and RMSE of linear, exponential, and polynomial models for LDL were 0.959, 0.016, and 0.127; 0.989, 0.004, and 0.064; and 0.998, 0.001, and 0.038, respectively, and the 2nd-order polynomial model was selected with respect to its *R*^2^, MSE, and RMSE values. For triglyceride, the 2nd-order polynomial model was selected with respect to its *R*^2^, MSE, and RMSE values of 0.991, 0.012, and 0.107.

To verify the calibrated models, it was important to compare observed against predicted total cholesterol level with corresponding goodness of fit, *R*^2^ = 0.999. The modeled total cholesterol values for *Costus afer* cotreatment at 0 mg/kg, 750 mg/kg, 1500 mg/kg, and 2250 mg/kg were 3.713, 3.021, 2.499, and 2.147, while the observed values were 3.720, 3.000, 2.520, and 2.140, respectively. The modeled HDL values for *Costus afer* cotreated groups of male albino rats at 0 mg/kg, 750 mg/kg, 1500 mg/kg, and 2250 mg/kg were 0.328, 0.426, 0.524, and 0.622, while the observed values were 0.340, 0.400, 0.540, and 0.620, respectively. The modeled LDL values for *Costus afer* cotreated groups of male albino rats at 0 mg/kg, 750 mg/kg, 1500 mg/kg, and 2250 mg/kg were 2.412, 01.846, 1.455, and 1.239, while the observed values were 2.420, 1.820, 1.480, and 1.230, respectively. The predicted triglyceride values for *Costus afer* cotreated groups of male albino rats at 0 mg/kg, 750 mg/kg, 1500 mg/kg, and 2250 mg/kg were 2.134, 1.658, 0.5152, and 0.616, while the observed values were 2.110, 1.730, 1.080, and 0.640, respectively. Hence, each model can be valued for predicting the applicable dependent variable for a given independent variable (*Costus afer*) at constant Pb, Cd, and Hg concentrations as used in this study.

With these models, it becomes unnecessary to repeat the lipid profile experiments carried out in the LDHMM treatment with *Costus afer* aqueous leaves extract (CALE) at 0, 750, 1500, and 2250 mg/kg. These models are useful in forecasting the residual values of lipid profile parameters of male albino rats at any treatment dose with *Costus afer* to a high precision at constant Pb, Cd, and Hg concentrations as used in this study. These models will help to reduce time wastage and resources in the treatment of LDHMM induced toxicity using *Costus afer*.

## 5. Conclusion

It is proven by this present study that, even at low doses, heavy metals can alter the serum lipid profile and hematological parameters of male albino rats after long term exposure. The heavy metal mixture treated rats had higher values of total cholesterol, LDL, triglycerides, and VLDL with lower HDL values compared to the normal control rats and rats cotreated with *Costus afer*. The dose dependent effect of *Costus afer* on the hematological and serum lipid profiles of heavy metal mixture exposed rats may suggest a beneficial mechanism in cardiovascular disease.

## Figures and Tables

**Table 1:** Effect of *Costus afer* on the feed intake (g) and fluid intake (ml) and food efficiency ratio (%) of male albino rats treated with metal mixture.

Treatment/weeks	^*∗*^1–4 wks		^*∗*^5–8 wks		^*∗*^9–12 wks		Feed efficiency ratio % (FER)
	Feed intake (g)	Fluid intake (ml)	Feed intake (g)	Fluid intake (ml)	Feed intake (g)	Fluid intake (ml)	
Deionized H_2_O (only)	221.44 ± 1.42^a^	434.54 ± 2.16^a^	222.45 ± 1.42^a^	439.39 ± 4.47^a^	226.36 ± 5.43^b^	439.45 ± 8.50^a^	0.398 ± 1.42^a^
Metal mixture (only)	205.94 ± 2.48^c^	409.80 ± 5.19^b^	192.60 ± 4.45^b^	357.21 ± 30.94^b^	98.23 ± 11.23^a^	306.46 ± 20.8^c^	0.397 ± 2.48^c^
Metal mixture + 750 mg/kg	202.88 ± 1.07^c^	400.59 ± 23.81^b^	207.43 ± 4.29^a^	409.80 ± 5.19^ab^	213.90 ± 2.85^b^	422.45 ± 3.38^b^	0.320 ± 1.07^c^
Metal mixture + 1500 mg/kg	207.43 ± 4.29^b^	416.66 ± 3.33^a^	213.90 ± 2.85^a^	418.67 ± 2.89^ab^	221.44 ± 1.42^b^	424.54 ± 2.16^b^	0.502 ± 4.29^b^
Metal mixture + 2250 mg/kg	222.45 ± 1.42^a^	417.50 ± 9.36^a^	223.86 ± 1.66^a^	424.54 ± 2.16^a^	234.61 ± 10.79^b^	439.39 ± 4.47^a^	0.469 ± 1.42^a^

^*∗*^Values are expressed as (mean ± SD, *N* = 4). In each column, values with different superscripts (a, b, and c) are significantly different from each other (*p* < 0.05) and those with the same superscripts are not significantly different.

**Table 2:** Effect of *Costus afer* on hematological parameters of male albino rats treated with metal mixture.

Treatment	T.WBC	NEU	LYM	MON	EOS	BAS	RBC	HGB	PCV	MCV	MCH	MCHC	PLT
Deionized H_2_O (only)	6.7 ± 1.87^a^	63.4 ± 8.44^c^	27.3 ± 6.81^a^	5.0 ± 3.03^b^	3.3 ± 1.58^b^	1.1 ± 0.32^b^	5.2 ± 0.19^d^	13.4 ± 1.51^c^	41.4 ± 3.76^d^	79.4 ± 8.31^a^	25.7 ± 2.96^a^	32.8 ± 5.84^a^	199.0 ± 40.99^a^
Metal mixture (only)	15.0 ± 2.02^d^	28.4 ± 4.32^a^	69.8 ± 4.59^d^	1.1 ± 0.34^a^	0.4 ± 0.05^a^	0.3 ± 0.08^a^	3.1 ± 0.15^a^	8.6 ± 1.07^a^	25.2 ± 3.14^a^	81.7 ± 9.54^ab^	28.0 ± 3.23^a^	34.8 ± 6.77^a^	219.0 ± 43.70^a^
Metal mixture + 750 mg/kg	12.2 ± 1.41^c^	35.2 ± 2.05^a^	62.3 ± 2.19^c^	1.3 ± 0.08^a^	0.7 ± 0.07^a^	0.6 ± 0.13^a^	3.4 ± 0.19^b^	10.0 ± 0.85^ab^	30.9 ± 1.05^b^	90.6 ± 6.92^b^	29.3 ± 2.74^a^	32.3 ± 2.28^a^	210.0 ± 18.37^a^
Metal mixture + 1500 mg/kg	9.6 ± 0.42^b^	43.4 ± 2.39^b^	52.7 ± 2.29^b^	1.8 ± 0.11^a^	1.1 ± 0.27^a^	0.9 ± 0.21^b^	4.1 ± 0.30^c^	11.8 ± 1.69^bc^	36.1 ± 1.66^c^	89.5 ± 8.85^ab^	29.2 ± 5.57^a^	32.6 ± 4.73^a^	206.0 ± 20.74^a^
Metal mixture + 2250 mg/kg	6.8 ± 1.22^a^	61.0 ± 8.54^c^	28.9 ± 7.46^a^	5.3 ± 1.73^b^	3.6 ± 0.75^b^	1.1 ± 0.36^b^	5.3 ± 0.21^d^	13.4 ± 1.79^c^	41.2 ± 2.02^d^	78.4 ± 5.07^a^	25.5 ± 3.71^a^	32.4 ± 3.02^a^	204.0 ± 19.81^a^

^*∗*^Values are expressed as (mean ± SD, *N* = 5). In each column, values with different superscripts (a, b, and c) are significantly different from each other (*p* < 0.05) and those with the same superscripts are not significantly different.

**Table 3:** Loadings of lipid profile variables on significant principal components after Varimax rotation.

Lipid profile parameters	^*∗*^PC1	PC2	PC3
Cholesterol (mmol/L)	**0.9499**	0.0295	0.0206
HDL (mmol/L)	**0.8890**	0.0000	0.1110
LDL (mmol/L)	**0.9001**	0.0988	0.0011
Triglyceride (mmol/L)	**0.9369**	0.0577	0.0054
VLDL (mmol/L)	**0.9369**	0.0577	0.0054
Eigenvalue	4.6128	0.2437	0.1435
Variability (%)	92.2564	4.8736	2.8700
Cumulative (%)	92.2564	97.1300	100.0000

^*∗*^Bold figures are highly correlated coefficients and thus are the principal factors.

**Table 4:** Concentration of heavy metals (mg/kg) on blood of male albino rats treated with metal mixture.

Treatment	Cadmium (Cd)	Mercury (Hg)	Lead (Pb)
Deionized H_2_O (only)	0.002 ± 0.001^a^	0.006 ± 0.003^a^	1.129 ± 0.087^a^
Metal mixture (only)	0.091 ± 0.007^d^	0.518 ± 0.021^e^	125.901 ± 15.069^d^
Metal mixture + 750 mg/kg CA	0.043 ± 0.006^c^	0.273 ± 0.028^d^	74.401 ± 9.615^c^
Metal mixture + 1500 mg/kg CA	0.024 ± 0.009^b^	0.163 ± 0.046^c^	35.745 ± 11.037^b^
Metal mixture + 2250 mg/kg CA	0.003 ± 0.003^a^	0.037 ± 0.016^ab^	5.037 ± 1.757^a^

^*∗*^Values are expressed as (mean ± SD). Values in the same column with different superscripts are significantly different from each other (*p* < 0.05) and those with the same superscripts in the same column are not significantly different, where CA = *Costus afer*.

**Table 5:** Summary of regression models for total cholesterol (TC) in serum.

Model type	Equation	*R* ^2^	MSE	RMSE
Exponential	*y* = 3.666*e*^−2*E*−0*x*^	0.995	0.003	0.055
Linear	*y* = −0.000*x* + 13.628	0.978	0.015	0.122
^*∗*^Polynomial (2^nd^ order)	*y* = 2*E* − 07*x*^2^ − 0.001*x* + 3.713	0.999	0.001	0.031

^*∗*^The best model with respect to *R*^2^, MSE, and RMSE values, where *y* = concentration of the total cholesterol and *x* = *Costus afer* dose.

**Table 6:** Model equations for serum lipid profile parameters.

Parameters	Model type	Model equations	*R* ^2^	MSE	RMSE
HDL	Linear	*y* = 0.000*x* + 0.328	0.978	0.001	0.023
LDL	Polynomial (2^nd^ order)	*y* = 2*E* − 07*x*^2^ − 0.000*x* + 2.411	0.998	0.001	0.038
TG	Linear	*y* = −0.000*x* + 2.149	0.990	0.006	0.079

*y* = concentration of the parameter analyzed, *x* = *Costus afer* dose, HDL = high-density lipoprotein, LDL = low-density lipoprotein, and TG = triglyceride, MSE = mean squared error, and RMSE = root mean squared error.

## Data Availability

No data were used to support this study.
